# Immunohistochemical over expression of p53 in head and neck Squamous cell carcinoma: clinical and prognostic significance

**DOI:** 10.1186/s13104-018-3547-7

**Published:** 2018-07-03

**Authors:** Atif Ali Hashmi, Zubaida Fida Hussain, Shumaila Kanwal Hashmi, Muhammad Irfan, Erum Yousuf Khan, Naveen Faridi, Amir Khan, Muhammad Muzzammil Edhi

**Affiliations:** 1 Liaquat National Hospital and Medical College, Karachi, Pakistan; 2 CMH Multan Institute of Medical Sciences, Multan, Pakistan; 3 Kandahar University, Kandahar, 3802 Afghanistan; 4 Brown University, Providence, RI USA

**Keywords:** Head and Squamous cell carcinoma, Oropharyngeal Squamous cell carcinoma, p53, Areca nut, Gutka

## Abstract

**Objective:**

Immunohistochemical over expression of p53 is considered as a marker of poor prognosis in many cancers. Therefore, we aimed to evaluate immunohistochemical overexpression of p53 in 121 cases of head and neck Squamous cell carcinoma and its association with various clinicopathologic features and survival.

**Results:**

Total 66.1% (80 cases) expressed positive p53 expression, 34% (29 cases) revealed no p53 expression, while focal positive p53 expression was noted in 9.9% (12 Cases). Moreover, high p53 expression (> 70%) was noted in 26.4% (32 cases), while 19% (23 cases) showed 51–70% p53 expression. On the basis of intensity of p53 staining; strong p53 expression was noted in 39.7% (48 cases), while 24.8% (30 cases) and 10.7% (13 cases) revealed intermediate and weak p53 expression respectively. Significant association of p53 intensity of expression with extranodal extension and higher tumor grade (grades II and III) was noted. p53 is useful prognostic biomarker in head and neck Squamous cell carcinoma and therefore we suggest that more large scale studies are needed to evaluate its prognostic significance in our population.

**Electronic supplementary material:**

The online version of this article (10.1186/s13104-018-3547-7) contains supplementary material, which is available to authorized users.

## Introduction

Despite advancements in cancer treatment, head and neck Squamous cell carcinoma (HNSCC) remains significant cause of morbidity and mortality worldwide, and hence 5 years survival rate is still below 50% [1, 2]. Owing to the widespread use of chewing tobacco and areca nut (pan/gutka) in South-Asia, oropharyngeal Squamous cell carcinoma (OSCC) is on a rise in this part of the world [3]. Oral carcinogenesis is a multistep process involving multiple proto-oncogenes and tumor suppressor genes including p16, cyclin D1, p53 and EGFR. TP53 is a tumor suppressor gene located on chromosome 17p. Mutations of TP53 gene is one of the most common event in human carcinogenesis. As mutated protein is not easily digestible, therefore it accumulates inside the cancer cell leading to immunohistochemical overexpression. p53 overexpression in OSCC is considered a marker of poor prognosis [4]. Moreover, p53 overexpression may result in decreased sensitivity of tumor cells to chemotherapeutic drugs [5]. Therefore, we aimed to evaluate immunohistochemical overexpression of p53 in our cases of HNSCC and its association with various clinicopathologic features and survival so that therapeutic protocols could be devised for loco-regional population.

## Main text

### Patients and methods

This was a retrospective study in which, 144 cases of HNSCC excision specimens were identified from previous records of pathology department. All patients had elective surgeries at Liaquat National hospital, Karachi from January 2008 till December 2013 over a period of 7 years. The approval of the study was taken from research and ethical review committee of the institution. Informed written consent was taken from all of the patients. Hematoxylin and eosin stained slides of all cases and paraffin blocks of 77 cases were recruited and new sections were cut when felt necessary. Slides of all cases were evaluated by two senior histopathologists independently and pathologic characteristics like tumor type, grade, tumor-stage, nodal-stage, lymphovascular invasion and perineural were interpreted. Clinical records of 57 patients were available and are thus reviewed from institutional records to evaluated patients age, smoking, alcohol and gutka/pan use history, history of radiation and chemotherapy. Moreover, representative tissue blocks of 121 cases were available for p53 immunohistochemistry.

### Immunohistochemistry

p53 IHC was performed using DAKO EnVision method using DAKO anti-human p53 protein, clone DO-7 according to manufacturers protocol. Nuclear staining for p53 was both quantitatively and qualitatively evaluated. Intensity of staining was grouped into no staining (0), weak (1+), intermediate (2+), strong (3+) while percentage of positively stained cells were calculated as a continuous variable. Intermediate to strong staining in more than 10% cancer cells was taken as positive while weak to intermediate staining in less than 10% tumor cells was interpreted as focal positive (Additional file 1: Figure S1). No staining in cancer cells was taken as negative. Moreover, p53 immunostaining was also categorized according to percentage of staining cells into different groups.

### Statistical analysis

Statistical package for social sciences (SPSS 21) was used for data compilation and analysis. Mean and standard deviation were evaluated for quantitative variables. For qualitative variables, frequency and percentage were calculated. Chi square was applied to determine association. *P* value of ≤ 0.05 was considered as significant. Statistical power of each variable was calculated by using software PASS version 11. Power of each tested variable is mentioned in Tables 2 and 3.

### Demographic profile of patients

Mean age of the patients included in our study was found to be 51.28 ± 12.14. Most common age group was between 31 and 50 years. Male gender was more common. Oral cavity was the most common origin of SCC found in our study (96 cases). High tumor stage (T3/T4) was noted in 25% (37 cases). Mean tumor size was 3.26 ± 1.67 cm while mean tumor depth was found to be 1.14 ± 0.78 cm. High nodal stage (N2/N3) was seen in 34.4% cases. 85 cases revealed keratinizing phenotype, while 11.1% cases were of high grade (grade III). Perineural and lymphovascular invasion was seen in 13.9 and 1.4% cases respectively as represented in Table 1.

**Table 1:** Clinicopathologic features of Squamous cell carcinoma head and neck (n = 144)

Characteristic	Frequency	Percentage (%)
Age (years)^a^	51.28 ± 12.14	
Age groups (years)		
≤ 30	6	4.2
31–50	72	50
> 50	66	45.8
Gender		
Male	106	73.6
Female	38	26.4
Histological subtypes		
Non-keratinizing	20	13.9
Keratinizing	85	59
Non-keratinizing with maturation	39	27.1
Histologic grade		
Grade-I	39	27.1
Grade-II	89	61.8
Grade-III	16	11.1
Location of tumor		
Oral cavity	96	66.7
Lip	3	2.1
Tongue	39	27.1
Soft palate	6	4.2
Tumor size (cm)^a^	3.26 ± 1.67	
Tumor stage		
T1	35	24.3
T2	72	50
T3/T4	37	25.7
Tumor depth (cm)^a^	1.14 ± 0.78	
Depth of invasion (cm)		
< 2	121	84
≥ 2	23	16
Nodal stage		
N0	76	52.8
N1	20	13.9
N2a	0	0
N2b	43	29.9
N2c	4	2.8
N3	1	0.7
Extra nodal extension		
Not present	109	75.7
Present	35	24.3
Lymphovascular invasion		
Not present	142	98.6
Present	2	1.4
Perineural invasion		
Not present	124	86.1
Present	20	13.9
Radiation (n = 86)		
Yes	35	60.3
No	23	39.7
Chemotherapy (n = 86)		
Yes	34	58.6
No	24	41.4
Recurrence (n = 86)		
Yes	33	56.9
No	25	43.1
History of pan (n = 57)		
Yes	34	59.6
No	23	40.4
History of smoking (n = 57)		
Yes	4	7
No	53	93
History of alcohol (n = 57)		
Yes	1	1.8
No	56	98.2

^a^Mean ± SD

### Immunohistochemical expression of p53 in head and neck Squamous cell carcinoma

Total 66.1% (80 cases) expressed positive p53 expression, 34% (29 cases) revealed no p53 expression, while focal positive p53 expression was noted in 9.9% (12 Cases). Moreover, high p53 expression (> 70%) was noted in 26.4% (32 cases), while 19% (23 cases) showed 51–70% p53 expression. 11–50 and < 10%/no expression was noted in 19% (23 cases) and 35.5% (43 cases) respectively. On the basis of intensity of p53 staining; strong p53 expression was noted in 39.7% (48 cases), while 24.8% (30 cases) and 10.7% (13 cases) revealed intermediate and weak p53 expression respectively. Tables 2 and 3 represent association of p53 expression with various clinicopathologic parameters in HNSCC. Significant association of p53 intensity of expression with extranodal extension and higher tumor grade (grades II and III) was noted. Association with those clinicopathological parameters with statistical power of less than 50 were not included in Tables 2 and 3.

**Table 2:** Association of p53 over expression categories with clinic pathologic parameters of head and neck Squamous cell carcinoma

	n (%)					*P* value	Power of test (%)
	≤ 10% (n = 43)	11–50% (n = 23)	51–70% (n = 23)	> 70% (n = 32)	Total (n = 121)		
Age group (years)							
≤ 30	3 (7)	0 (0)	1 (4.3)	1 (3.1)	5 (4.1)	0.502	37.12
31–50	23 (53.5)	8 (34.8)	10 (43.5)	17 (53.1)	58 (47.9)		
> 50	17 (39.5)	15 (65.2)	12 (52.2)	14 (43.8)	58 (47.9)		
Gender							
Male	36 (83.7)	15 (65.2)	16 (69.6)	22 (68.8)	89 (73.6)	0.299	33.12
Female	7 (16.3)	8 (34.8)	7 (30.4)	10 (31.3)	32 (26.4)		
Depth of invasion (cm)							
< 2	36 (83.7)	17 (73.9)	23 (100)	26 (81.3)	102 (84.3)	0.056	54.7
≥ 2	7 (16.3)	6 (26.1)	0 (0)	6 (18.8)	19 (15.7)		
Nodal stage							
N0	26 (60.5)	16 (69.6)	12 (52.2)	13 (40.6)	67 (55.4)	0.258	65.64
N1	8 (18.6)	1 (4.3)	3 (13)	6 (18.8)	18 (14.9)		
N2a	0 (0)	0 (0)	0 (0)	0 (0)	0 (0)		
N2b	9 (20.9)	5 (21.7)	7 (30.4)	11 (34.4)	32 (26.4)		
N2c	0 (0)	0 (0)	1 (4.3)	2 (6.3)	3 (2.5)		
N3	0 (0)	1 (4.3)	0 (0)	0 (0)	1 (0.8)		
Histological subtypes							
Non-keratinizing	3 (7)	1 (4.3)	4 (17.4)	10 (31.3)	18 (14.9)	0.100	71.32
Keratinizing	28 (65.1)	15 (65.2)	12 (52.2)	14 (43.8)	69 (57)		
Non-keratinizing with maturation	12 (27.9)	7 (30.4)	7 (30.4)	8 (25)	34 (28.1)		
Histologic grade							
Grade-I	13 (30.2)	11 (47.8)	5 (21.7)	4 (12.5)	33 (27.3)	0.065	74.37
Grade-II	24 (55.8)	11 (47.8)	13 (56.5)	25 (78.1)	73 (60.3)		
Grade-III	6 (14)	1 (4.3)	5 (21.7)	3 (9.4)	15 (12.4)		
Perineural invasion							
Not present	39 (90.7)	22 (95.7)	21 (91.3)	23 (71.9)	105 (86.8)	0.051	69.81
Present	4 (9.3)	1 (4.3)	2 (8.7)	9 (28.1)	16 (13.2)		

Chi square test was applied

P-value ≤ 0.05 considered as significant

**Table 3:** Association of p53 expression intensity with clinicopathologic parameters of head and neck Squamous cell carcinoma

	n (%)					P-value	Power of test (%)
	No intensity (n = 30)	Weak (n = 13)	Intermediate (n = 30)	Strong (n = 48)	Total (n = 121)		
Age group (years)							
≤ 30	3 (10)	0 (0)	1 (3.3)	1 (2.1)	5 (4.1)	0.445	43.47
31–50	15 (50)	8 (61.5)	11 (36.7)	24 (50)	58 (47.9)		
> 50	12 (40)	5 (38.5)	18 (60)	23 (47.9)	58 (47.9)		
Gender							
Male	25 (83.3)	11 (84.6)	22 (73.3)	31 (64.6)	89 (73.6)	0.269	38.17
Female	5 (16.7)	2 (15.4)	8 (26.7)	17 (35.4)	32 (26.4)		
Nodal stage							
N0	17 (56.7)	9 (69.2)	22 (73.3)	19 (39.6)	67 (55.4)	0.053	82.44
N1	8 (26.7)	0 (0)	2 (6.7)	8 (16.7)	18 (14.9)		
N2a	0 (0)	0 (0)	0 (0)	0 (0)	0 (0)		
N2b	5 (16.7)	4 (30.8)	6 (20)	17 (35.4)	32 (26.4)		
N2c	0 (0)	0 (0)	0 (0)	3 (6.3)	3 (2.5)		
N3	0 (0)	0 (0)	0 (0)	1 (2.1)	1 (0.8)		
Extranodal extension							
Not present	22 (73.3)	11 (84.6)	28 (93.3)	29 (60.4)	90 (74.4)	0.008	81.53
Present	8 (26.7)	2 (15.4)	2 (6.7)	19 (39.6)	31 (25.6)		
Histologic grade							
Grade-I	6 (20)	7 (53.8)	12 (40)	8 (16.7)	33 (27.3)	0.046	74.91
Grade-II	20 (66.7)	4 (30.8)	14 (46.7)	35 (72.9)	73 (60.3)		
Grade-III	4 (13.3)	2 (15.4)	4 (13.3)	5 (10.4)	15 (12.4)		

Chi square test was applied

P-value ≤ 0.05 considered as significant

## Discussion

In the present study, we found that 66.1% of HNCC revealed p53 overexpression; moreover significant association of p53 was noted with extranodal extension and tumor grade, which are key prognostic factors of HNSCC, thus proving the prognostic significance of this biomarker.

p53 overexpression in HNSCC varies in different parts of the world, owing to divergent risk factors and pathogenesis of the disease. Kerdpon et al. reported a positive association of alcohol use with p53 over expression and negative association with betal nut and tobacco [6]. P53 expression in HNSCC ranges from 25 to 90%. Dragomir et al. reported 31% p53 expression [7], whereas, Gonzalez-Moles revealed 57.7% expression of p53 [8]. On the other hand, 85.6% p53 expression was reported in a study from India [5], 63.3% expression was noted in a research conducted in Brazilian population [9], while as low as 28.5% expression was noted in a study conducted in Iran [10]. Varied expression of p53 in HNSCC may be due to different use of techniques, methods of interpretation or due to difference in ethnicity and risk factors involved in HNSCC pathogenesis. Previous literature also showed association of p53 expression with tumor grade and other histologic features. Dave et al. in a study involving 40 cases of HNCC found a significant association of p53 expression with tumor grade and other histologic parameters like degree of keratinization [11]. Although we found significant association of p53 expression with tumor grade, however association with other histologic parameters was not noted. Although no significant association was noted between p53 expression and other clinicopathological parameters in our study, however as the statistical power for these associations was less than 50% therefore, no conclusion could be derived.

Many studies have proved the prognostic significance of p53 in HNCC, owing to association of p53 overexpression with overall survival, recurrence, high tumor grade and T and N stage [5, 8, 12]. Conversely, a meta-analysis involving 174 studies revealed no association of p53 overexpression as a marker of poor prognosis [13]. We found a significant association of intensity of p53 expression with extranodal extension and tumor grade, with a large sample size and significant statistical power the associations are important; therefore we suggest more large scale studies to evaluate prognostic significance of p53 expression in HNSCC and its association with disease free survival in loco-regional population.

## Limitation

Major limitation of the study was that, this was a single center data, however as it’s a major tertiary care hospital of the province therefore the results may have major clinical implications. Furthermore, recurrence status of patients was not available to evaluate association p53 expression with disease free survival.

## Additional file

**Additional file 1: Figure S1.** p53 expression in oral Squamous cell carcinoma.

## Abbreviations

HNSCC: head and neck Squamous cell carcinoma
OSCC: oropharyngeal Squamous cell carcinoma
