# Supplementary figures and images for: Immunohistochemical over expression of p53 in head and neck Squamous cell carcinoma: clinical and prognostic significance
Source: BMC Res Notes. 2018 Jul 3;11:433. doi: 10.1186/s13104-018-3547-7 (PMC6029369; doi:10.1186/s13104-018-3547-7)

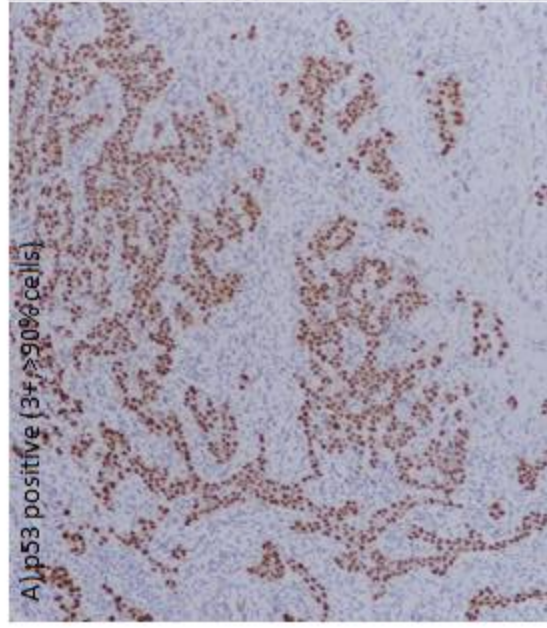

A) p53 positive (3+ >90% cells)

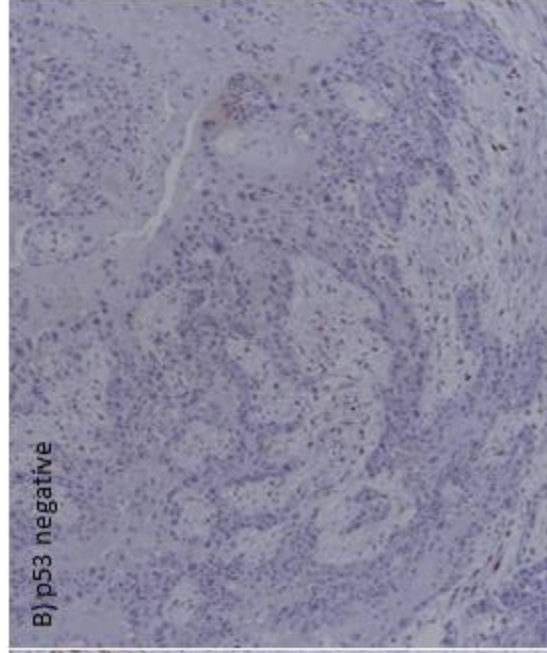

B) p53 negative

Supplement: Supplementary file 1 — Additional file 1: Figure S1. p53 expression in oral Squamous cell carcinoma. [file 13104_2018_3547_MOESM1_ESM.pdf]
